# Knowledge and Informed Decision-Making about Population-Based Colorectal Cancer Screening Participation in Groups with Low and Adequate Health Literacy

**DOI:** 10.1155/2016/7292369

**Published:** 2016-04-20

**Authors:** M. L. Essink-Bot, E. Dekker, D. R. M. Timmermans, E. Uiters, M. P. Fransen

**Affiliations:** ^1^Department of Public Health, Academic Medical Center, University of Amsterdam, P.O. Box 22660, 1100 DD Amsterdam, Netherlands; ^2^Department of Gastroenterology and Hepatology, Academic Medical Center, University of Amsterdam, P.O. Box 22660, 1100 DD Amsterdam, Netherlands; ^3^Department of Public and Occupational Health, EMGO Institute for Health and Care Research, VU University Medical Center Amsterdam, P.O. Box 7057, 1007 MB Amsterdam, Netherlands; ^4^National Institute for Public Health and the Environment (RIVM), P.O. Box 1, 3720 BA Bilthoven, Netherlands

## Abstract

*Objective*. To analyze and compare decision-relevant knowledge, decisional conflict, and informed decision-making about colorectal cancer (CRC) screening participation between potential screening participants with low and adequate health literacy (HL), defined as the skills to access, understand, and apply information to make informed decisions about health.* Methods*. Survey including 71 individuals with low HL and 70 with adequate HL, all eligible for the Dutch organized CRC screening program. Knowledge, attitude, intention to participate, and decisional conflict were assessed after reading the standard information materials. HL was assessed using the Short Assessment of Health Literacy in Dutch. Informed decision-making was analyzed by the multidimensional measure of informed choice.* Results*. 64% of the study population had adequate knowledge of CRC and CRC screening (low HL 43/71 (61%), adequate HL 47/70 (67%), *p* > 0.05). 57% were informed decision-makers (low HL 34/71 (55%), adequate HL 39/70 (58%), *p* > 0.05). Intention to participate was 89% (low HL 63/71 (89%), adequate HL 63/70 (90%)). Respondents with low HL experienced significantly more decisional conflict (25.8 versus 16.1; *p* = 0.00).* Conclusion*. Informed decision-making about CRC screening participation was suboptimal among both individuals with low HL and individuals with adequate HL. Further research is required to develop and implement effective strategies to convey decision-relevant knowledge about CRC screening to all screening invitees.

## 1. Introduction

Colorectal cancer (CRC) is one of the most common causes of cancer-related death in the world [1]. Identification of preclinical precursors and preclinical stages of CRC by screening can contribute to a decrease of CRC-related mortality [2]. In Netherlands, a nationwide organized population-based CRC screening program has been rolled out since 2014 [3]. All individuals between 55 and 75 years of age will receive a standard information package and a test-kit with an immunochemical faecal occult blood test (iFOBT) by postal mail. The information package includes an invitation letter and an information leaflet about CRC and the CRC screening program; additional information can be found on a central website [4]. The information package explicitly aims at enabling individuals to make an autonomous, informed decision about CRC screening participation [4, 5]. According to Marteau et al. [6], an informed decision to participate is based on adequate decision-relevant knowledge and a positive attitude towards participation.

As with any cancer screening, CRC screening has inherent disadvantageous side effects, including false positive and false negative iFOBT results [7]. Follow-up colonoscopy is associated with a low risk of potentially serious harm. Therefore, enabling informed decision-making about cancer screening participation has been recommended [8]. Screening invitees need to be able to deliberate on the pros and cons of their own screening participation based on correct knowledge and to make a decision that is genuinely consistent with their values. Recommendations in the EU Guidelines 2010 reflect the wide consensus view that people who are invited to use CRC screening services should have access to accurate and understandable information that reflects current epidemiological evidence about CRC screening, its potential contribution to reducing the risk of CRC death in the population, and information about its risks and limitations [7]. Informed decision-making about CRC screening participation in the population is a challenge, due to the complexity of any cancer screening program and, with regard to CRC screening programs as organized in Netherlands and also in the UK, the fact that people decide on participation without the initial support of a professional [7].

Informed decision-making about CRC screening participation may be especially challenging in groups with low health literacy, that is, inadequate skills to access, understand, and apply information to make informed decisions about their health [9]. In Netherlands, at least 25% of the adult Dutch population has been estimated to have inadequate health literacy [10, 11]. Socioeconomic inequalities in organized population-based CRC screening participation have been demonstrated repeatedly [12, 13] and inadequate HL was shown to be an important mediator of lower CRC screening participation among groups with a lower socioeconomic status [14–16]. Uninformed decision-making in healthcare is generally more prevalent among poorly educated and low health literate groups [17–20]. However, the relationship between health literacy and informed decision-making about CRC screening participation was not investigated before. Therefore, we analyzed and compared decision-relevant knowledge and informed decision-making after reading the standard information package of the Dutch nationwide CRC screening program between potential screening invitees with low and adequate HL. We also assessed decisional conflict about CRC screening participation.

## 2. Methods

### 2.1. Study Design and Population

In November and December 2013 (i.e., before the official start of the national CRC screening program), 1500 members (55–75 years of age) of an online test panel of Netherlands Institute for Health Services Research (NIVEL) were invited to participate [21]. This panel consists of approximately 6,750 individuals aged 18 years or older who agreed to participate in online surveys on a regular basis. Each individual member receives a questionnaire around three times a year (by post or through the Internet).

**Figure 1:**
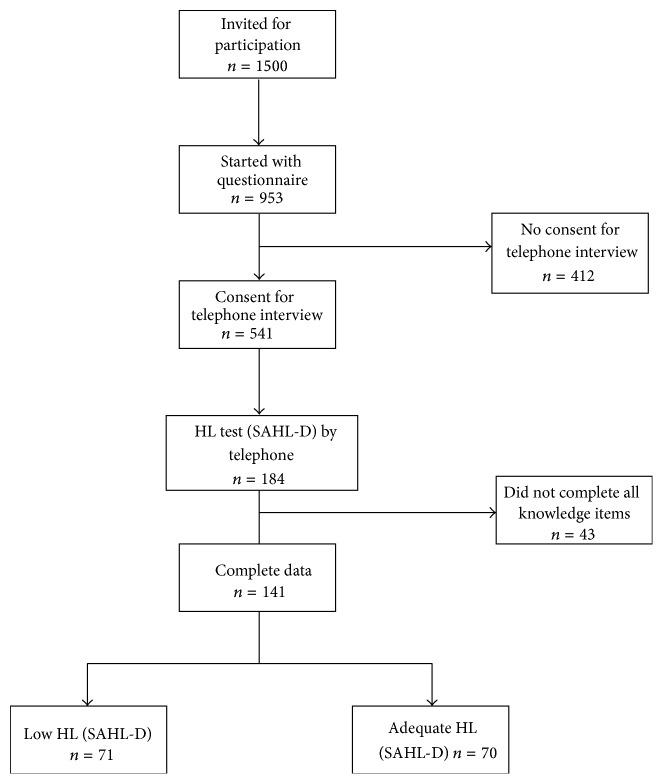
Flowchart of the selection of the study population.

The 641 panel members who gave permission to be contacted by telephone for assessment of their health literacy level were sent the online questionnaire (see below) that was returned by 541 panel members (response rate: 84%). For the current analysis, questionnaire data were complemented by a performance-based HL test (see below) [22] in a sample of the study population through telephone interviews. Health literacy data were available for 184/541 respondents. To include groups with low and adequate HL of comparable size, we purposively selected panel members by educational level in the last half of telephone assessments. For the present analysis, we used HL and questionnaire data of 141 responders that provided complete data for the knowledge questionnaire items before and after reading the information materials (see Figure 1).

According to the Dutch Medical Research Involving Human Subjects Act, this study did not require medicoethical approval, as was confirmed in writing by the medical ethical committee of the Academic Medical Center Amsterdam, Netherlands (May 13, 2013). We took every possible precaution to protect the privacy of the respondents.

### 2.2. Online Questionnaire

Respondents were provided a link to the standard information package of the Dutch CRC screening program, that is, announcement letter and the test package including an invitation letter, a reply form, information leaflet, and an instruction leaflet. They were instructed to read this and to complete the online questionnaire afterwards. Using the information materials during completion of the questionnaire was allowed.

#### 2.2.1. Knowledge

Knowledge was assessed with 16 items: 6 items about CRC in general and 10 items about CRC screening [23]. All items were statements with response options “correct,” “incorrect,” or “don't know.” Responses that correctly identified a given statement as “correct” or “incorrect” were scored as 1; responses that incorrectly identified a statement as “correct” or “incorrect” or the use of the “don't know” option were classified as 0. Summing of the scores resulted in individual knowledge scores ranging from 0 (lowest possible score) to 16 (best possible score).

Following the original scoring developed by Denters et al. [23] a knowledge score was considered to reflect adequate decision-relevant knowledge if at least two-thirds of knowledge statements had been correctly identified (total knowledge score >11) under the condition that at least half of the items on general CRC knowledge (general knowledge score >4) and at least two-thirds of the CRC screening specific items (screening specific knowledge score >7) were correctly identified.

#### 2.2.2. Attitude

Attitude towards own participation in CRC screening was assessed with 10 items using 5-point Likert scales that were used in a Dutch study on decision-making in CRC screening [24]. Respondents were asked to indicate if participation in CRC screening for themselves would be a good-bad idea, frightening-not frightening, reassuring-not reassuring, self-evident-not self-evident, important-unimportant, wise-unwise, desirable-undesirable, pleasant-unpleasant, harmful-not harmful, and useful-not useful. Scores were summed, resulting in total attitude scale ranging from 10 to 50. Scores below 30 indicated a negative attitude; scores of 30 and above indicated a positive attitude.

#### 2.2.3. Intention to Participate

Intention to participate in the CRC screening program was measured by one item: “Will you take part in the CRC screening program when you will be invited?” measured on a 5-point Likert scale (certainly, probably, perhaps, probably not, and certainly not).

#### 2.2.4. Informed Decision-Making

Informed decisions about CRC screening participation, according to Marteau et al.'s multidimensional measure of informed choice, were based on a combination of decision-relevant knowledge, attitude, and intention to participate in the CRC screening program [6]. An informed decision to participate is based on adequate knowledge, a positive attitude, and a positive intention to participate. An informed decision not to participate is based on adequate knowledge, a negative attitude, and a negative intention to participate.

#### 2.2.5. Decisional Conflict

Decisional conflict was assessed with the Dutch version of the Decisional Conflict Scale, consisting of 10 items with response options of “yes” (scored as 0), “don't know” (2), or “no” (4) [25, 26]. The sum of the scores was multiplied by 2.5 following the original scoring as developed by O'Connor [25], resulting in a final score between 0 (no decisional conflict) and 100 (extreme decisional conflict).

#### 2.2.6. Demographics

We assessed self-reported gender, age (years), educational level (afterwards classified into low, medium, and high), ethnic origin (defined by country of birth, following Statistics Netherlands [27], ethnic Dutch-ethnic non-Dutch), and language commonly used (Dutch or another language).

### 2.3. Health Literacy Assessment

Health literacy was assessed by telephone using the Short Assessment of Health Literacy in Dutch (SAHL-D), a test based on word recognition and comprehension in the health domain [22]. During the telephone interview respondents received an email with the SAHL-D attached in pdf format. After they opened the file they were asked to read each word aloud and to choose one of the possible meanings from the options that were presented on the same page. Following cut-off points defined in the validation study of the SAHL-D, those with <55 out of 66 correct answers were considered as having inadequate HL.

### 2.4. Data Analysis

We used descriptive statistics to characterize the study groups and their scores and Chi-square tests to analyze differences in knowledge, decisional conflict, and IDM scores between the groups with low and high HL. Differences in mean knowledge and decisional conflict were tested by ANOVA. All analyses were conducted using SPSS for Windows version 20.

## 3. Results

### 3.1. Characteristics of the Study Population

Of the 141 respondents, 71 participants had low HL and 70 participants had adequate HL. Women were relatively overrepresented (64%) in the adequate HL group. Respondents with adequate HL were generally higher educated than the group with low HL, but among the group with low HL 13% reported a high educational level. 96% of the respondents were of Dutch ethnic origin and all used Dutch as spoken language most of the time (Table 1).

### 3.2. Knowledge

Table 2 shows that respondents with low HL classified 2 out of 16 knowledge items significantly less often correctly than respondents with adequate HL (*p* < 0.05). For four other knowledge statements, the differences between low and adequate HL were borderline significant (0.05 ≤ *p* < 0.10). Seven items were classified correctly by more than 90% of respondents in both HL groups. Two items received correct classifications by approximately 50% of the respondents or less across the HL groups. One of these, the item “if a person has CRC, there is a 100% chance the stool test will detect it” reflects the concept of false negative results of the initial test. The largest difference between respondents with low and adequate HL was found for the item on screening participation in the absence of symptoms. 100% of the respondents with adequate HL correctly responded that CRC screening is useful in the absence of symptoms of CRC, compared to 91% of the respondents with low HL. The mean knowledge was significantly lower among respondents with low HL (13.21 versus 13.94; *p* = 0.03; see Table 3). Knowledge was adequate in 64% of all respondents (low HL 61%, adequate HL 67%; *p* = 0.41).

### 3.3. Informed Decision-Making

Of all respondents, 89% had the intention to participate in the CRC screening program. 57% of all individuals would make an informed participation decision (low HL 55%, adequate HL 58%; *p* = 0.70) (Table 3).

### 3.4. Decisional Conflict

The average Decisional Conflict Scale score was 21.1, but respondents with low HL exhibited an average DCS score of 25.8 compared to 16.1 among respondents with adequate HL (*F* = 14.03; *p* = 0.00).

## 4. Discussion

After reading the standard information package on the Dutch CRC screening program, 64% of the study population had adequate knowledge of CRC and CRC screening. The differences in knowledge scores between respondents with low and adequate HL were small and statistically insignificant. Similarly, informed decision-making about CRC screening participation was present in 57%, with no significant differences between low and adequate HL groups. However, potential screenees with low HL experienced significantly more decisional conflict. The latter finding is in line with a study from the USA that found lower levels of informed choice and more decisional conflict about cancer screening among groups of lower educational attainment levels, although this study was not conducted in the context of an organized screening program [28].

Comparison of knowledge about CRC and CRC screening between low and adequate HL groups at item level showed deficiencies in knowledge about the possibility of false negative initial screening results, both in the low and in the adequate HL groups. Knowing that screening participation may result in false negative test results seems essential for understanding the consequences of screening participation and for informed decision-making. In a previous study among participants to a pilot CRC screening program, Denters et al. [23] also found deficient knowledge about the possibility of false negative results. Educational inequalities in CRC screening uptake have been extensively documented in the USA and in the UK [13, 14]. Kobayashi (2013) showed that limited health literacy is a barrier to participation in England's national CRC screening program, and von Wagner et al. [12] showed that low health literacy had a direct impact on information seeking and was associated with lower self-efficacy in performing the CRC screening test. Smith et al. [19] showed that lower educational level and lower literacy levels are associated with more difficulties making informed choices about participation in bowel screening in Australia. In Netherlands, Denters et al. [23] found that 91% of CRC screening invitees in the third round of an implementation pilot preceding the national CRC screening program made an informed decision, with 92% having sufficient knowledge. The difference with 57% informed choices in our study population may be explained by the fact that Denters et al. analyzed responses of invitees for a third screening round, whereas our study population was screening naïve. Because we found only 58% informed choice among individuals with adequate HL, the difference does not seem attributable to oversampling of respondents with low HL in our study.

The finding of suboptimal levels of decision-relevant knowledge across HL levels after reading the information package may increase doubts about the feasibility of adequately informing potential screenees through written information only, as confirmed by the ASCEND trial [29, 30]. One of the causes may be the inherent complexity of balanced information about cancer screening. Especially among groups with a lower educational attainment level, information processing is more difficult, and not every individual is capable of digesting epidemiological evidence on risks and benefits [15, 19, 31]. Expert-defined epidemiological knowledge about the population-based pros and cons of CRC screening may also be perceived as less relevant for the decision-making process [32]. What exactly constitutes an informed decision? Should it be more consistent with how people make complex decisions and give meaning to health risks in daily life [33–36]? Some have argued that the current focus on cognitive decision-making about CRC screening participation may even inhibit rather than promote informed decision-making [35].

### 4.1. Strengths and Limitations

The direct comparison of respondents with low and adequate HL, which was not previously published in relation to informed decision-making in CRC screening, is a strength of this study. The performance-based testing of individual HL can also be considered as strength, because the association between self-perceived HL and performance-based measurement is often quite low [37]. Our study also had its limitations. The research was conducted in 2013, the year preceding the start of the national colorectal screening program in Netherlands (January 1, 2014). Therefore measuring actual uptake was not an option. The study design was cross-sectional, using the assumption that health literacy is a stable characteristic over time in this age group and that the measurement of performance was not influenced by completion of the questionnaire beforehand. Because we oversampled respondents with low HL, the research group is not to be considered as representative of the Dutch general population. The respondents were a selective sample anyway, as reflected by a 100% positive attitude towards CRC screening. Most likely, study participants who were interested in screening were overrepresented in the study sample, as often the case in studies on informed choice in cancer screening [38].

### 4.2. Implications

The current results of suboptimal decision-relevant CRC screening knowledge add to previous evidence that information strategies consisting of only written materials may be insufficient, even among individuals with adequate health literacy who are potentially interested in CRC screening. Especially among lower educated groups, informed decision-making may require additional support for those who need it because of limitations in information processing capabilities in this group [39]. Further research may focus on evidence-based development and implementation of strategies to adequately support low HL groups in their decision-making about screening participation.

## Figures and Tables

**Table 1:** Demographics of the study population (*n* = 141).

	All respondents *n* = 141 (%)	Low health literacy *n* = 71 (%)	Adequate health literacy *n* = 70 (%)
Gender			
Men, *n* (%)	60 (42)	35 (49)	25 (36)
Women, *n* (%)	81 (57)	36 (51)	45 (64)
Age, mean ± SD	66.7 ± 5.3	67.6 ± 4.5	65.8 ± 5.8
Education level^*∗*^			
Low, *n* (%)	31 (23)	27 (39)	4 (6)
Intermediate, *n* (%)	77 (57)	33 (48)	44 (66)
High, *n* (%)	28 (20)	9 (13)	19 (28)
Ethnic origin^*∗∗*^			
Ethnic Dutch, *n* (%)	135 (96)	68 (99)	67 (97)
Non-Dutch, *n* (%)	3 (4)	1 (1)	2 (3)

^*∗*^5 missing: 2 in low HL group, 3 in adequate HL group.

^*∗∗*^3 missing: 2 in low HL group, 1 in adequate HL group.

**Table 2:** Knowledge about CRC and CRC screening among respondents with low and adequate health literacy, after reading the standard information package (*n* = 141).

	Correct responses				*p* value^*∗*^ Chi-square
	Low HL (total *n* = 71)		High HL (total *n* = 70)		
	*n*	%	*n*	%	
*CRC-specific items*					
A mass screening programme can detect CRC before it becomes symptomatic (correct)	69	97	68	97	0.99
CRC has a better chance of survival when detected in an early stage (correct)	70	99	70	100	0.32
Persons can die from CRC when not treated on time (correct)	60	84	58	83	0.79
CRC can be hereditary (correct)	32	45	36	51	0.45
CRC is one of the most prevalent cancers in the Netherlands (correct)	52	73	49	70	0.67
Younger persons have a higher chance of being diagnosed with CRC than older persons (incorrect)	**58**	**82**	**66**	**94**	**0.02**
*CRC screening specific items*					
In the absence of symptoms, participation is not useful (incorrect)	**65**	**91**	**70**	**100**	**0.01**
The presence of blood in stool can be a sign of CRC (correct)	66	93	68	97	0.25
The stool test has to be repeated every two years (correct)	66	93	67	96	0.48
If the stool test detects blood, there is a 100% change of CRC (incorrect)	*65*	*91*	*69*	*99*	*0.06*
If a person has CRC, there is a 100% chance the stool test will detect this (incorrect)	*34*	*48*	*45*	*64*	*0.05*
If the stool test detects blood, a follow-up investigation is necessary to check for the presence of CRC (correct)	70	99	69	99	0.99
The follow-up investigation (a colonoscopy) is in almost 100% of cases correct in detecting CRC (correct)	*53*	*75*	*61*	*87*	*0.06*
If the colonoscopy detects precursors of CRC, these can almost always be removed in the same procedure (correct)	55	77	56	80	0.71
After removal of precursor lesions, regular checkups of the bowel are not necessary (incorrect)	63	89	58	83	0.32
Participation in the screening program is obligatory for person between the ages of 55 and 75 (incorrect)	*60*	*84*	*66*	*94*	*0.06*

^*∗*^ *p* < 0.05 in bold; 0.05 ≤ *p* < 0.10 in italics.

**Table 3:** Informed decision-making about CRC screening participation among respondents with low and adequate health literacy (*n* = 141).

	All respondents *n* = 141	Respondents with low health literacy *n* = 71	Respondents with adequate health literacy *n* = 70	*p* value Chi-square (Pearson)	*p* value ANOVA (*F* test)
Total knowledge of CRC and CRC screening, mean ± SD	**13.57 ± 1.95**	**13.21 ± 2.20**	**13.94 ± 1.61**		**0.03 (F 5.09)**
Knowledge					
Adequate, *n* (%)	90 (64)	43 (61)	47 (67)	0.41	
Inadequate, *n* (%)	51 (36)	28 (39)	23 (33)		
Attitude towards CRC screening					
Positive, *n* (%)	129 (100)	60 (97)	67 (100)	0.14	
Negative, *n* (%)	0 (0)	2 (3)	0 (0)		
Missing	*12*	*9*	*3*		
Intention to participate in CRC screening					
Positive, *n* (%)	126 (89)	63 (89)	63 (90)	0.81	
Negative, *n* (%)	15 (11)	8 (11)	7 (10)		
Attitude-uptake consistency^1^					
Consistent, *n* (%)	116 (90)	56 (90)	60 (90)	0.89	
Not consistent, *n* (%)	13 (10)	6 (10)	7 (10)		
Missing	12	9	3		
Informed decision-making					
Informed choice, *n* (%)	73 (57)	34 (55)	39 (58)	0.70	
No informed choice, *n* (%)	56 (43)	28 (45)	28 (42)		
Missing	12	9	3		
Decisional conflict, mean ± SD	**21.12 ± 15.54**	**25.82 ± 17.96**	**16.05 ± 10.37**		**0.00 (F 14.03)**

*p* < 0.05 in bold.

^1^Attitude-uptake consistency means a combination of a negative attitude with an intention not to participate or a positive attitude with an intention to participate. Inconsistency means a negative attitude and an intention to participate or a positive attitude and an intention not to participate. Attitude-uptake inconsistency is one of the elements of uninformed choice.
